# Optimism

**Published:** 1906-02

**Authors:** 


### EDITORIAL NOTES AND COMMENTS.

The Business office of THE JOURNAL-RECORD is No. 1007-1008 Century Building.

The Editorial office is 318-319-320 The Prudential.

Address all Business communications to Dr. M. B. Hutchins, Mgr.

Make remittances payable to THE ATLANTA JOURNAL-RECORD OF MEDICINE.

On matters pertaining to the Editorial and Original Communications address Dr. Bernard Wolff, Atlanta.

Reprints of original articles will be furnished at cost price. Requests for the same should always be made *on the manuscript*.

We will present, post-paid, on request, to each contributor of an original article, twenty (20) marked copies of THE JOURNAL-RECORD containing such article.

### OPTIMISM.

OBJECTION has often been brought against the untrustworthiness of the reports of results achieved from the use of new drugs and other therapeutic remedies. Putting aside immature conclusions, errors of diagnosis and inadequate time-tests, which are not vicious in motive, there are many instances of this character which are without reasonable doubt deliberate falsifications and published with the evident intent of furthering some one's interest. It is needless to say that this does not include the publications of charlatans, but refers to alleged ethical preparations or instruments.

One of the most flagrant examples of this species of deception which has come to notice of late years is contained in the literature of the "therapeutic lamp" manufacturers and consists in case reports by physicians who are using this remedy. There is no question of the efficacy of phototherapy when judiciously applied in selected cases, but there is no surer way of bringing discredit upon the method than by making claims for it which are preposterous and absurd. According to these reports, which are very near kin to the newspaper "testimonial," chronic diarrhea, chronic bronchitis, cystitis and enlargement of the prostate, appendicitis, peritonitis, eczema and "kidney troubles" readily yield to a few applications of the light.

Contrast these reports with the results obtained by Finsen from the use in lupus of an 80,000 candle-power arc light. Finsen secured favorable results after months of continuous treatment, while with a 25 candle-power incandescent lamp Finsen himself might have been spared to the world and cured of his "kidney trouble" in less time than it would have taken him to heal a patch of lupus as big as his thumbnail.

The following illustrations are taken at random from among a large number of reports, all favorable: "Chronic neurasthenia for years, tumor of abdomen, 'canker' of mouth and stomach. Sixteen treatments resulting as follows: Tumor almost disappeared, 'canker' all gone, patient has ravenous appetite and is so well that she has hired herself to a dressmaker and is working from six to twelve hours a day."

"An old chronic case of gall-stone colic, ovarian and bladder troubles, neuralgia everywhere; a total wreck and almost bedridden. Also had chronic catarrh, deafness increasing with each attack of gall-stone colic. The first lamp treatment relieved the neuralgia, the second took away all soreness from the parts. The deafness is vastly improved. She is now a well woman. Has no gall-stone colic and is truly happy and grateful." And well might she be!

Subacute peritonitis, cured in one application of the light.

Suppuration of the testicle, cured by off-and-on treatment. "Patient and *his relatives* very grateful for saving the testicle, which was in frightful peril at the hands of a most estimable surgeon."

Occipital headaches for many years. Ten treatments made her perfectly well.

Another "old chronic case" of gastritis with recurrent nodule on tongue, arousing suspicion of cancer, but proving to be "canker." Eleven treatments caused a disappearance of the canker and she is now admitted by all to be the happiest woman of the "beautiful city" in which she resides, wherever that is. Is it truly the "Beautiful City, far away in the Kingdom of God"?

Chronic bronchitis with "threatened" consumption fled before eight light exposures.

The most remarkable case, however, as the writer of the report truly says, was that of incipient cataract. The writer's enthusiasm leads him to say that "To see these cataracts dissolve from week to week and to hear the patient say from day to day how the vision was improving was most exhilarating and encouraging to both physician and patient—and then to finally see clearly all the posterior of each eye was a climax that surpassed everything and crowned our efforts with perfect success."

Now, we do not attempt to deny the plain, unvarnished truth of these certificates. The reader may reach his own conclusions by his own methods of reasoning.

*Are* they true? "Have I the law on my side if I say, No?"

---

THE physicians of Elbert county met in Elberton, December 5th, and formed a county medical society on the standard plan. The following officers were elected: President, Dr. A. S. J. Stovall, Elberton; vice-president, Dr. Amos C. Smith, Coldwater; secretary and treasurer, Dr. William A. Matthews, Elberton, and censors, Drs. Joseph E. Johnson, L. Pope Eberhardt, and A. B. Matthews, all of Elberton.

---

THE regents of the State University, Ann Arbor, have decided to condemn the west end of the old medical building. No classes will be heard in its halls after the close of the first semester.

